# A little history goes a long way toward understanding why we study consciousness the way we do today

**DOI:** 10.1073/pnas.1921623117

**Published:** 2020-03-13

**Authors:** Joseph E. LeDoux, Matthias Michel, Hakwan Lau

**Affiliations:** ^a^Center for Neural Science, New York University, New York, NY 10003;; ^b^Department of Psychology, New York University, New York, NY 10003;; ^c^Department of Psychiatry, New York University Langone Medical School, New York, NY 10003;; ^d^Department of Child and Adolescent Psychiatry, New York University Langone Medical School, New York, NY 10003;; ^e^Emotional Brain Institute, Nathan Kline Institute, Orangeburg, NY 10962;; ^f^Consciousness, Cognition & Computation Group, Université Libre de Bruxelles, B1050 Bruxelles, Belgium;; ^g^Center for Mind, Brain and Consciousness, New York University, New York, NY 10003;; ^h^Department of Psychology, University of California, Los Angeles, CA 90095;; ^i^Brain Research Institute, University of California, Los Angeles, CA 90095;; ^j^Department of Psychology, University of Hong Kong, Hong Kong;; ^k^State Key Laboratory of Brain and Cognitive Sciences, University of Hong Kong, Hong Kong

**Keywords:** consciousness, unconscious, amnesia, blindsight, split brain

## Abstract

Consciousness is currently a thriving area of research in psychology and neuroscience. While this is often attributed to events that took place in the early 1990s, consciousness studies today are a continuation of research that started in the late 19th century and that continued throughout the 20th century. From the beginning, the effort built on studies of animals to reveal basic principles of brain organization and function, and of human patients to gain clues about consciousness itself. Particularly important and our focus here is research in the 1950s, 1960s, and 1970s involving three groups of patients—amnesia, split brain, and blindsight. Across all three groups, a similar pattern of results was found—the patients could respond appropriately to stimuli that they denied seeing (or in the case of amnesiacs, having seen before). These studies paved the way for the current wave of research on consciousness. The field is, in fact, still grappling with the implications of the findings showing that the ability to consciously know and report the identity of a visual stimulus can be dissociated in the brain from the mechanisms that underlie the ability to behave in a meaningful way to the same stimulus.

Figuring out how our brains make our conscious experiences is one of the most interesting and challenging scientific topics today. Clarification of the mechanisms involved is crucial for a deeper understanding of human nature and the problems that we face as individuals and societies. Knowledge of the history of current issues about consciousness places us in a better position to make scientific progress on this topic.

Despite the central importance of consciousness to human mental life, scientific psychology has had a complex relationship with it (1–3). Many early psychologists were introspectionists and prized consciousness. Behaviorists later banned it from the field. Cognitivists, upon dethroning behaviorism, focused on information processing rather than subjective experience, keeping consciousness within reach but seldom touching it.

Today, the scientific study of consciousness is a vibrant area of research in psychology and neuroscience. Influential papers by Francis Crick and Christof Koch in the early 1990s (4–6) are often credited for instigating this turn of events (7–10). In particular, they are credited for having defined an empirical approach to consciousness—by focusing on visual awareness, progress could be made on consciousness since so much is known about the brain’s visual system.*

The Crick and Koch papers were indeed important for stimulating enthusiasm for research on consciousness and the brain in mainstream psychology and neuroscience. However, this was hardly the beginning of scientific interest in and research on consciousness. In the 1960s and 1970s, studies of split-brain, blindsight, and amnesia patients laid the conceptual foundations for later work on consciousness. Of note is the fact that even then most of this work focused on visual consciousness because of the progress that had been made in understanding the visual system (13, 14). Additionally, consciousness and the brain were the subject of a number of scientific conferences starting in the 1950s that were attended by leading researchers in psychology and brain science (15–17). Furthermore, theories about what consciousness is and how it relates to the brain were proposed by a number of prominent researchers long before the 1990s, including Karl Lashley (18–20), Wilder Penfield (21), Donald Hebb (22, 23), Roger Sperry (24–27), Sir John Eccles (28), George Miller (29), Lord Brain (30), Michael Gazzaniga (31), Leon Festinger and coworkers (32), George Mandler (33), Tim Shallice (34), and Michael Posner and coworkers (35) among others.

Our goal in this article is to provide a historical account of some of the key research findings and theories about consciousness that have been overshadowed by more recent history. The focus will be on consciousness as subjective experience rather than on other meanings, such as the ability to be awake and responsive to external stimuli.

## The Foundations of Consciousness Research in the Late 19th and Early 20th Centuries

Although our emphasis will be on the mid-20th century, this period must be contextualized by the fact that research on brain and consciousness, like many other topics in psychology and brain science, began in the late 19th century. This was a time when psychological questions were driven by philosophical understanding of the mind, which was often equated with consciousness. As a result, research on brain and behavior naturally considered the role of consciousness in behavioral control by the brain.

As is still common today, these early researchers explored the effects of surgical ablation or electrical stimulation of brain areas (1, 36). Several studies demonstrated that decorticated animals could exhibit high degrees of behavioral flexibility (37, 38). These observations led to a debate as to whether the behavioral responses of decorticated animals were driven by unconscious sensitivity or conscious sensations, and whether having a cerebral cortex was necessary for having conscious experiences (36).

The main arguments in favor of the view that the cerebral cortex is necessary for consciousness came from David Ferrier’s pioneering electrical stimulation studies (39). He is mainly known for his work on stimulations of the motor cortex of animals. But Ferrier (39) also demonstrated that stimulations of parietal and temporal lobes caused animals to behave as if they were having visual, tactile, auditory, or olfactory sensations, while stimulations of subcortical sensory areas, including the optic thalamus, did not. Ferrier (39) concluded that activity in the cerebral cortex may be sufficient for eliciting conscious experiences, while subcortical processes control complex behaviors nonconsciously (36).

Ferrier felt that it was essential to study consciousness in humans, warning that researchers cannot rely on behavioral appearances alone in animals: “the plaintive cry elicited by pinching the foot of a rabbit may be merely a reflex phenomenon, not depending on any true sense of pain” (39). By contrast, studies of humans can use verbal reports to assess “consciousness of impressions” (39).

Observations involving human neurological patients indeed began shaping views of consciousness during this time. The most influential work in this area was perhaps that of Ferrier’s friend and mentor, John Hughlings Jackson, who observed that epileptic seizures arising from focal regions in the brain are sometimes accompanied by alterations in conscious experience (40). He proposed that consciousness was the highest level of cerebral organization and that mind involved interactions between conscious and unconscious processes (41). The importance of Ferrier and Hughlings Jackson at the end of the 19th century cannot be overstated. They greatly influenced the next generation of researchers who would study consciousness, and also impacted Sigmund Freud’s writings about consciousness and the unconscious.

Simultaneously, in Germany in the late 19th century, the field of experimental psychology was also emerging as a scientific discipline, one in which philosophical questions about the mind, especially consciousness, began to be addressed in laboratory studies using the experimental methods of physiology. The studies by Ferrier and his contemporaries were crucial to this development. Also important was the work of Gustav Fechner, who introduced psychophysical methods for rigorously relating the physical properties of stimuli with psychological experiences. Of additional note is Hermann von Helmholtz, who worked on the physiology of sensation and proposed the notion that conscious perception involves unconscious inferences, foreshadowing the idea that consciousness depends on nonconscious processing. While these researchers worked on psychological topics, the first researcher officially considered to be an experimental psychologist was the German scientist Wilhelm Wundt (1). In the United States, William James claimed that honor.

Consciousness was a central concern of these various 19th-century researchers. However, it also began to be used gratuitously as an account of human behavior (1). By the early 20th century, consciousness was often simply assumed to underlie behavior. This point is thrown into relief by the growing influence of Sigmund Freud’s views about unconscious aspects of mind (42).

Somewhat separately, Darwin’s theory of evolution had promoted a wave of cross-species studies of behavior in the late 19th and early 20th centuries (43). Although Ferrier had warned about the perils of attributing mental states to nonhuman species, Darwin’s followers, like Darwin himself, readily called on human-like emotions and other conscious mental states to account for the behaviors of animals (43, 44).

In response to the interpretive excesses of human psychology and the rampant anthropomorphism in animal psychology, in 1913 John Watson (45) proposed that a scientific psychology must be based on observable events (stimuli and responses) and not on presumptions about mental states. The result was the behaviorist movement, which essentially banned subjective experience from the field of experimental psychology throughout much of the first half of the 20th century.

Meanwhile, the medical sciences operated outside of the concerns of academic psychology and were unaffected by behaviorists’ constraints. For example, the physiologist Charles Sherrington (46) continued stimulation work in animals in the footsteps of Ferrier. He is considered the father of modern neurophysiology and is especially well known for his work on spinal reflexes (46). However, for our story, of particular importance is that he wrote about consciousness and that he trained Wilder Penfield.

In the 1930s and 1940s, Penfield (47) performed pioneering studies of consciousness in humans. He applied electrical stimulation to the brains of awake epileptic patients for the purpose of localizing key areas involved in language and thought so that these regions could be avoided when he subsequently surgically removed areas with seizure activity (47). While Ferrier and Sherrington could only speculate about whether conscious experiences were elicited by electrical stimulation of cortical areas in monkeys, Penfield and coworkers (47, 48) were able to obtain verbal reports from patients about their subjective experiences. His work provided compelling evidence for a role of the cerebral cortex in conscious experiences (49).

In sum, research in the 19th century initiated several themes that are prominent today: the mind has conscious and nonconscious aspects, conscious experience depends on nonconscious processes, and the cerebral cortex plays a key role in consciousness.

## A New Approach to Brain and Behavior

The standard approaches now taken in research on brain and behavior today had their origin in the work of Karl Spencer Lashley (50). He completed his PhD in 1914 working on the behavior of an invertebrate organism, the hydra, at Johns Hopkins. There, he met John Watson, who published his initial proclamation about behaviorism (45) during Lashley’s time at Hopkins.

Through Watson, Lashley was introduced to the work of Shepherd Ivory Franz (51), the first researcher to use the new conditioning methods of behaviorism in conjunction with brain lesions to study the brain mechanisms of behavior. He designed behavioral tasks to test specific brain functions to reveal effects of brain damage that were not apparent from mere observation alone. Lashley used this approach in his landmark studies that sought to find the “engram,” the storage mechanism of memory (52–54). Although Lashley and Watson remained friends for many years, they disagreed on one major topic. In 1923, when behaviorism was just getting going, Lashley published an article chiding behaviorists for their rigid views about consciousness (20).

The Franz/Lashley approach to the study of brain and behavior acquired a name when Lashley used term “neuropsychology” in a 1936 lecture to the Boston Society of Psychiatry and Neurology (55). In subsequent years, the field of neuropsychology thrived using the Franz/Lashley approach in animal models but also, in human studies of patients with natural lesions from neurological diseases or surgical lesions made in an effort to treat neurological problems. Many of the key figures in the scientific history of research on brain and behavior in the 20th century, including many of the researchers we will discuss below, are represented in Lashley’s scientific family tree^†^ (for additional information see *SI Appendix*, Box 1).

In the 1950s, just as cognitive science was beginning to replace behaviorism, Lashley (54) published an important paper that emphasized how consciousness emerges from nonconscious information processing. This idea echoed Ferrier and Helmholtz and was foundational in early cognitive science (2, 29, 56) and also became underlying assumption in the later history of consciousness research that we pursue below.

## Animal Neuropsychology Paved the Way

Neuropsychological research on animals is of interest to our discussion of consciousness, not because it necessarily revealed anything about consciousness per se. The work was instead important because it provided a neuroanatomical and conceptual foundation that guided the design and interpretation of studies of human patients.

The most important institute for neuropsychological research on animals in the 1940s was the Yerkes Primate Center in Florida, which was directed by Lashley. Researchers there were trained in the Franz/Lashley approach and used specific behavioral tasks to test specific brain functions. When the neurosurgeon Karl Pribram took over the directorship at Yerkes shortly after the end of World War II, he continued the behavioral approach established by Lashley but with added neurosurgical sophistication.^‡^ The field of animal neuropsychology flourished during Pribram’s decade-long rein at Yerkes. Young researchers who would come to be the face of the field cut their scientific teeth at Yerkes under Pribram’s guidance.

The main method used at the time was surgically placed lesions, and the Yerkes group studied the effects of lesions within all major lobes of the cerebral cortex, and also of subcortical areas, such as the amygdala. In the tradition of Lashley, animals were studied using specific behavioral tasks designed to test hypotheses about brain function. While many important discoveries were made, of note for our purposes here were studies that clarified which regions of the temporal lobe contributed to distinct aspects of the Kluver–Bucy syndrome.

Heinrich Kluver and Paul Bucy published a seminal paper in 1937 (57, 58). Kluver was interested in brain mechanisms underlying hallucinations induced by mescaline. He observed that monkeys receiving mescaline often smacked their lips, a symptom that occurs when humans with temporal lobe epilepsy have seizures and report hallucinations (59). Bucy, a human neurosurgeon, was recruited to produce lesions of the temporal lobe in monkeys. The animals were found to exhibit a suite of striking behavioral changes, including increased timidity, hyperorality, and hypersexuality. Kluver and Bucy referred to the condition as one of “psychic blindness.” The animals were not blind, but visual stimuli lost their meaning—snakes and people were no longer threatening to them; they tried to eat objects previously known to be inedible, and they attempted to have sex with other species. Although similar findings had been reported much earlier (60), as we will see, the paper by Kluver and Bucy was extremely influential in shaping brain and behavior research that followed World War II in the United States, where basic science had been put on hold during the war effort.

The psychic blindness phenomenon or what neurologists called “visual agnosia” was the subject of much work at Yerkes. This was pursued using visual discrimination learning to create stimuli with complex visual meaning. Studies by Mortimer Mishkin and coworkers (13, 61, 62) showed deficits in such tasks following damage to subareas of the temporal lobe. Specifically, damage to either the lateral temporal lobe (which is connected to the visual cortex) or the ventral temporal lobe (which is connected to the hippocampus) impaired behavioral performance. One implication was that complex visual processing came to be understood as extending beyond the occipital lobe into the temporal lobe. Additionally, because the tasks depended on learning and memory, the work became especially important in understanding how memories are formed and stored in the brain, especially via the hippocampus, as discussed below.

Other work by Miskhin and coworkers (63–66) implicated specific areas of prefrontal cortex in tasks that tax short-term memory or what is now called “working memory” (67, 68). Building on this research, later behavioral studies of prefrontal cortex in monkeys were the foundation for understanding the role of prefrontal cortex in human working memory (69–71). This is important here because, from the 1970s onward, many researchers have equated consciousness with the contents of a short-term memory system (33, 35, 72) or with the availability of information for executive planning systems (73). Working memory and the prefrontal cortex are still central to cognitive theories of consciousness (74–78).

Mishkin went on to head the Laboratory of Neuropsychology at the National Institute of Mental Health, where he continued to follow-up on the questions raised by the Kluver–Bucy syndrome. Specifically, he and his colleagues pursued the role of the temporal lobe in perception, memory, and affective/emotional processing (79–82). The distinction between the ventral and dorsal streams of visual processing, crucial for consciousness research today, emerged from his laboratory (79), as did the key role of the perirhinal cortex as a link between the visual cortex and the hippocampus in memory formation (81).

Larry Weiskrantz, another member of Pribram’s group, also worked on the temporal lobe and visual memory (62) but additionally on the importance of the amygdala in the affective aspects of the Kluver–Bucy syndrome (83). From Yerkes, Weiskrantz moved to Cambridge and later became chair of experimental psychology at Oxford. One topic that he pursued after moving to England was the contribution of cortical areas to memory (84). However, the thrust of his career was defined by his work on blindsight (85), a phenomenon that is central to current debates about the nature of consciousness in humans.

Only a few examples of the output and implications of research done at the Yerkes laboratory in the 1950s were mentioned here, but it would be hard to overstate the importance of this group. These researchers paved the way for much future work on the brain mechanisms of perception, memory, emotion, and higher cognition, and also of consciousness.

## Human Neuropsychological Research Brought Consciousness into the Mainstream of Psychology and Neuroscience

Neuropsychological research on patients produced novel insights into brain and behavior, including the relation of consciousness to the brain. Studies of three groups of patients were especially important (78) and will be our focus below. These were amnesia patients (in whom natural or surgical lesions in the medial temporal lobe disrupted the ability to form and recall new memories), split-brain patients (in whom the two cerebral hemispheres were surgically separated to reduce the impact of intractable epilepsy), and blindsight patients (in whom visual cortex damage produced apparent blindness in the visual field opposite the lesion locus). In all three groups, findings demonstrated striking dissociations between what patients could do behaviorally and what they could consciously report. Other patient groups (coma, hemineglect, aphasia, prosopagnosia, and dyslexia) also exhibited dissociations between explicit knowledge and behavioral performance and thus contributed to the emergence of interest in consciousness (*SI Appendix*, Box 2) (86). However, amnesia, split-brain, and blindsight patients are focused on here because of their broad impact on the field.

### Amnesia.

The prevailing view through much of the first half of the 20th century was that memory is widely distributed in the brain rather than localized in a specific area. This was based, in part, on Lashley’s work suggesting that memory depended more on the amount of cortical tissue damaged than on the location of the damage, with different areas being “equipotential” in their ability to store memories (52, 54). The tides shifted in the 1950s.

A major figure in this sea change was Brenda Milner, a PhD student at McGill University. She was especially interested in memory and intellectual functions of the temporal lobe, but her work turned out to be particularly important for understanding the relation of memory to consciousness. Milner did her PhD research working under the renowned psychologist Donald Hebb, who trained with Lashley and wrote extensively about memory and behavior but also, about consciousness (22, 23). Milner was aware of the above-mentioned stimulation studies carried out by Penfield, who was head of neurosurgery at McGill. Hebb was owed a favor by Penfield, who had had a number of patients with temporal lobe removals since this is a major site of epilepsy, and Penfield agreed to let Milner study them.^§^ She tested 45 patients with temporal lobe damage on tasks assessing cognitive functions but mainly focused on the effects of such lesions on learning, especially visual learning, and memory.

Her thesis, published in 1954 (87), began with a detailed review of what was known about the functions of the temporal lobe from studies of monkeys and especially, of the effects of temporal lobe lesions on visual learning as this seemed particularly relevant to human visual memory. Milner relied heavily on work by Mortimer Mishkin (88), who studied visual discrimination in monkeys at McGill for his PhD before joining Pribram at Yerkes. Although Mishkin found that deep temporal lobe lesions involving the hippocampus impaired performance, he interpreted this effect as being caused by damage to nerve fiber pathways passing through the temporal lobe (88).

In her studies of Penfield’s patients, Milner used a variety of tests. From these, she concluded that, as in monkeys, the temporal lobe plays a key role in visual learning in humans. Following graduation, she remained at McGill and continued researching the psychological functions of temporal lobe. However, her most important finding was not on Penfield’s patients but on a patient operated on by William Scoville in Hartford, CT (89). This was patient HM, studies of whom revolutionized the research on memory (90).

The initial studies of HM were interpreted in terms of a general memory deficit, a so-called global amnesia. However, later work by Milner (91) and Suzanne Corkin (92) determined that HM and other amnesic patients retained the ability to learn and remember how to perform motor skills (for example, drawing objects while looking at their reversed reflection in a mirror). Over time, other examples of spared memory were identified, and it became clear that, in addition to motor skills, the patients could also learn cognitive skills (93), could form behavioral habits, and could develop Pavlovian conditioned responses (94). Extrapolating across these findings, Larry Squire and Neal Cohen (93) proposed in 1980 that the memory deficit resulting from temporal lobe damage was limited to declarative memory, memory that could be consciously experienced. For example, although the patients could learn motor skills and be conditioned, they could not consciously remember having recently acquired the skill or having been conditioned. Conscious memory came to be referred to by the designations “declarative” or “explicit,” and nonconscious memory came to be referred to as “procedural” or “implicit” (95, 96). Explicit memory itself was split into two subtypes: episodic and semantic (97).

HM and other patients with problems involving explicit memory had damage that included a relatively large region of the temporal lobe. Animal studies could be more precise in targeting specific subareas involved in explicit memory; these areas came to be known as the “medial temporal lobe memory system” (98). For example, studies by Mishkin and Murray (99) and Squire and Zola-Morgan (98) showed that the hippocampus, entorhinal cortex, parahippocampal region, and perihrinal cortex each contributed to the storage of new memories. With this knowledge, it was possible to find select cases that confirmed the contribution of different areas to different aspects of consciously accessible memory in humans (100, 101).

A trend in recent years involves recognition that prefrontal cortex plays an important role in the retrieval of explicit memories, including the conscious experience of the retrieved memories (102–105). Another recent trend has focused on how explicit memories are used to construct conscious simulations of future and other hypothetical experiences (106, 107). As we will see, evidence from all three patient groups hints at a role for prefrontal cortex in conscious experience

### Split-Brain Syndrome.

Split-brain surgery involves surgical section of the corpus callosum and other lesser cerebral commissures in an effort to help relieve intractable epilepsy. These pathways are composed of axons that interconnect corresponding areas in the two hemispheres. Noticing reports that, after recovery from surgery, such patients are remarkable in their lack of noticeable effects of the procedure, Roger Sperry, a brain researcher at Cal Tech, wondered about the actual function of the callosum. He initiated a series of studies in cats and monkeys to try to solve this mystery, which he called “one of the more intriguing and challenging enigmas of brain function” (108).

Sperry’s studies in animals with split-brain operations confirmed the clinical impression from humans. Thus, following damage to the corpus callosum, split-brain animals appeared rather ordinary. In the tradition of Lashley, his mentor at Yerkes, Sperry (108) and his colleagues designed specific experimental tasks to shed light on the function of the corpus callosum and other commissures.

In these studies, in addition to sectioning the various commissures, the optic chiasm was also sectioned in order to restrict the flow of visual inputs from each eye to the opposite hemisphere. As a first step, animals learned to perform a response for reinforcement. At this stage, one eye and hemisphere were trained and then tested. Subsequently, the occlusion was switched to the other eye to assess the other hemisphere. Animals that only received section of the optic chiasm performed well with each eye. However, when the commissures were also sectioned, the untrained eye and hemisphere could not perform. Nevertheless, the same hemisphere then had no trouble learning the problem on its own. Thus, learning is normally shared by the two hemispheres, but when the commissures are cut, the untrained hemisphere cannot access the memory. Many variations of these studies were performed in Sperry’s laboratory (31, 108, 109).

In the early 1960s, Sperry began collaborating with Joseph Bogen, a neurosurgeon in Los Angeles who was performing split-brain surgery in humans with intractable epilepsy. The patients were studied by Michael Gazzaniga, a graduate student in Sperry’s laboratory (31, 110). Because the optic chiasm was not part of this surgery, Gazzaniga had to find some other way to restrict visual stimuli to one hemisphere. Given that visual information in the right visual field is sent to the left hemisphere and that visual information in the left visual field is sent to the right hemisphere, he could project stimuli onto a screen and restrict which hemisphere received the inputs as long as the eyes were stationary. To prevent eye movements from having an effect, the stimuli were presented briefly (about 250 ms). He also designed specific tests tailored to the special properties of the human brain and in particular, issues that result from the lateralization of function.

For example, in most people, the ability to speak and understand spoken and written language is localized to the left hemisphere. People with typical brains can thus name common objects that appear in either the left or right visual field because visual information reaching the visual cortex in one hemisphere is communicated to the same area in the other hemisphere via the corpus callosum. While split-brain patients are able to give verbal reports about information presented to the right visual field and thus, the left hemisphere, they cannot name stimuli in the left half of visual space, thus seen by the right hemisphere. They can, however, respond nonverbally to the stimuli seen by the right hemisphere by pointing toward or grabbing objects with the left hand, which is preferentially connected to the right hemisphere. Similarly, when blindfolded, these subjects can name objects placed in their right hand (preferentially connected to the left hemisphere) but not objects placed in their left hand.

Although the right hemisphere of split-brain patients was not able to verbally report on its inner states, it could nevertheless respond nonverbally (for example, by pointing) to indicate that it has meaningfully processed visual stimuli. This led to the idea that, following split-brain surgery, each hemisphere not only has separate behavioral control capacities but possibly, separate mental systems—two conscious beings. The possibility of two minds, one in each hemisphere, was speculated about and much discussed in the scientific and popular literature (111–114). However, the extent of possible mentation in the right hemisphere was difficult to test in the absence of its ability to provide a verbal report.

In the early 1970s, Gazzaniga (115–119) began studies of a new group of patients operated at Dartmouth. Many of the basic findings about the isolation of perception, memory, and cognition in the two hemispheres were confirmed (115–119). One of these patients (referred to as PS) provided perhaps the first compelling evidence suggesting that dual minds could exist in split-brain patients. This patient had the ability to read with both hemispheres but could only speak with the left (115, 120). Although the right hemisphere could not speak, it could respond verbally to visual questions in the left visual field by using his left hand to select Scrabble letters. To the question, “Who are you?,” the left hand spelled his name, “Paul.” Also, to the question of his desired occupation, the left hand spelled “race car driver.” This was of particular interest since the left hemisphere said “draftsman” to the verbally stated question. Despite not being able to communicate, the two hemispheres shared personal identity (Paul) yet had different life ambitions.

The findings suggested that an isolated right hemisphere can have a separate conscious awareness of self and a vision of the future. More extensive studies by Gazzaniga (116, 118) and colleagues of subsequent patients, especially JW, also supported the dual-mental systems idea. A key unresolved question is whether all split-brain patients have dual consciousness or whether, in some, brain pathology leads to some compensatory reorganization and changes what the right hemisphere can do (*SI Appendix*, Box 3).

Another important outcome of this work was to suggest what role consciousness might have in our mental economy (78, 115–119, 121, 122). From the point of view of the left hemisphere, responses coming from the right hemisphere are generated nonconsciously. Studies involving the patient who could read via his right hemisphere were designed to elicit behavioral responses by presenting visual verbal commands to the left visual field. The experimenter then asked out loud, “Why did you do that.” The patient then responded via his left hemisphere with a verbal answer. The left hemisphere routinely took things in stride, telling a tale that made the responses make sense. For example, when the command to the right hemisphere was “stand up,” he (his left hemisphere) explained his action by saying, “Oh, I needed to stretch.” This was obviously pure confabulation since the left hemisphere was not privy to the information that instructed him to stand up.

To account for these findings, the theory of cognitive dissonance by Leon Festinger (123) was called on. The theory proposed that mismatches between what one expects and what actually happens create a state of inner discordance or dissonance. Because dissonance is stressful, it demands reduction. Thus, when the patient became aware that his body produced a response that “he” did not initiate, dissonance resulted, and the confabulation of a reason why the response occurred was a means of reducing dissonance. Today, “postdecision rationalization” is an active research topic that examines how people retroactively justify their decisions and actions in life (124, 125).

The narratives weaved by the left hemisphere were viewed as interpretations of situations and were proposed to be an important mechanism used by humans to maintain a sense of mental unity in the face of neural diversity (115–119). The narration/interpretation process was later proposed to depend on cognitive functions of prefrontal cortex related to working memory and to be consistent with cognitive theories of consciousness (78, 121, 122, 126).

### Blindsight.

Blindsight is the clinical condition that is most often discussed in the context of the contemporary science of consciousness. Damage to the primary visual cortex produces an apparent blindness in the visual field opposite to the lesion (85). Yet, when requested to do so, blindsight patients can make guesses about the identity or presence of visual stimuli presented to the “blind” field at accuracy levels that are well above chance. They are consciously blind but can “see” sufficiently to control behavior.

The existence of such residual vision following damage to primary visual cortex (V1) was reported in 1967 by Larry Weiskrantz and Nicholas Humphrey (127). A monkey (called Helen) with bilateral damage to the visual cortex could still respond to visual stimuli (blinking, reaching to stimuli, pupillary responses, and so on). Similar findings were also reported earlier in patients with damage to the occipital lobe by Riddoch (128) and Poppel et al. (129). However, both for patients and in monkeys, the subjective phenomenology was unclear.

Weiskrantz (85), who, as mentioned above, had been trained at Yerkes, made two important contributions to address the question of whether conscious experience can occur after V1 damage. First, he introduced what he called “commentary keys.” On every trial, after the patient made a forced choice answer regarding the stimulus, Weiskrantz asked them to press keys to indicate explicitly whether they had seen the stimulus consciously or were responding on some other basis. This may seem like a simple experimental procedure, but it reflected Weiskrantz’s open attitude toward studying subjective phenomenology and consciousness, which was contrary to the norms of experimental visual psychophysics at the time. Weiskrantz concluded that the patients’ above-chance guessing was subjectively unconscious. This led to his second key contribution: he coined the term “blindsight,” making it explicit that the phenomenon observed in these patients was about a selective impairment of conscious experience (85, 130).

Commentary keys were also used in monkeys with visual cortex lesions resulting in blindsight-like behavior. For example, from such studies, Stoerig and Cowey (131) proposed that it is likely that monkeys have visual phenomenal consciousness. Weiskrantz (85) noted that this is “easy to accept, but not to prove.” He argued that, since consciousness is not always necessary for human perception and behavior, evidence that animals produce appropriate behavioral responses to visual stimuli does not, on its own, necessarily qualify as evidence that they are conscious of what they see (85).

Even the interpretation of the human findings about consciousness was met with some skepticism, especially by empirically rigorous vision scientists (reviewed in ref. 132). To further address whether blindsight patients were truly unconscious of the stimuli or whether they meant they saw them poorly when they said they did not see them consciously, researchers showed that blindsight is qualitatively unlike weak, near-threshold vision (reviewed in ref. 132). Specifically, the detectability of stimuli is impaired in blindsight relative to what one may expect given the subjects’ performance in forced choice tasks. (Signal detection theory accounts of these psychophysical findings are in ref. 133.)

Similar psychophysical signatures have also been observed in monkeys with V1 lesions as well (134). This, in turn, addresses another concern, which is that the human patients’ lesion may not be complete (135). In monkeys, the lesions were surgically created and confirmed carefully, and therefore, the issue of incomplete lesion could be ruled out (136–138). This is consistent with the conclusion that the behavioral responses in blindsight are not due to spared cortex in V1.

Although blindsight is about vision, it also speaks to affective processes. In particular, it was found that patients can unconsciously detect emotional expressions in faces presented to the blind field (139). These findings further demonstrate the possibility of striking dissociations between conscious experience and depth of unconscious processing of complex stimuli. They also corroborate the view that the processes in the amygdala can be driven unconsciously and do not necessarily reflect conscious emotions (140).

What may be the neural basis of blindsight? It is known that some stimuli, such as motion, can elicit activity in extrastriate visual areas even in the absence of V1 (137, 138). The pathway continues to be mapped out in more detail by new studies, but the visual signal likely goes from the retina to subcortical areas, like the lateral geniculate nucleus, superior colliculus, and pulvinar, and from there directly to extrastriate areas, bypassing V1 (138). This leads to the question of why the patients are not visually conscious, given that there is activity in the visual (extrastriate) areas. An intuitive view may be that feedback to V1 is necessary (141). However, such a view would be incompatible with findings that patients without V1 may nonetheless sometimes have conscious visual experiences (142, 143).

Weiskrantz (85) suggested that projection of signals to the prefrontal cortex may be crucial for visual consciousness. Although the prefrontal cortex receives direct projections from extrastriate areas rather than V1 itself, the idea is that, when V1 is damaged, the dynamics of the signals in extrastriate areas may not allow for sufficiently normal propagation into the prefrontal cortex. This hypothesis has been confirmed in several neuroimaging studies (144, 145) in which the prefrontal cortex showed higher activity for conscious perception compared with blindsight within a single patient who had blindsight in only part of the visual field. This is also compatible with other findings in neuropsychology. Using what is sometimes called a double-lesion method, Nakamura and Mishkin (146) found that monkeys with unilateral frontal and parietal lesions, when combined with other ablations that blocked the information flow from the visual cortex to the frontal and parietal cortices in the remaining hemisphere, showed chronic “blindness”-like behavior. Therefore, apparently having an intact visual cortex is not enough for visual behavior unless it is connected to the remaining frontal and parietal cortices. As Weiskrantz suggested, signals to prefrontal cortex may be necessary for conscious awareness (85), at least in humans.

## Conclusions

1) The idea that research on consciousness could advance by focusing on vision was not a new idea in the 1990s. This was an implicit assumption underlying practically all of the work on consciousness since the late 19th century as well as throughout the 20th century, including but not limited to studies of patients with amnesia, split brains, and blindsight.
2) Findings from each of the three patient groups demonstrated profound dissociations between what the patients could report and what they could respond to behaviorally. These dissociations are conceptually important because the impairments are not in the general ability to process any information. They specifically involve an inability to subjectively report on conscious experiences.
3) Researchers from all three traditions—amnesia (106, 107), split brain (3, 117, 121, 140), and blindsight (76, 85, 147)—independently reached the conclusion that consciousness involves higher cognitive processes that depend, at least in part, on prefrontal cortex. This conclusion is consistent with contemporary cognitive theories of consciousness, including the global workspace theory (74, 53) and the higher-order theory (76–78, 148).

### Data Availability Statement.

There are no data associated with this manuscript.

## Supplementary Material

Supplementary File

## Footnotes

*Other researchers also helped stimulate interest in consciousness in the 1990s, including Antonio Damasio (11) and Gerald Edelman (12).

^†^https://neurotree.org/beta/tree.php?pid=119.

^‡^Information about Pribram’s life is available at http://karlpribram.com/karl-pribram-1919-2015/.

^§^Information is available at http://www.psych.ualberta.ca/∼gcpws//Milner/Biography/Milner_bio4.html.
