# Supplementary material for: A little history goes a long way toward understanding why we study consciousness the way we do today
Source: Proc Natl Acad Sci U S A. 2020 Mar 13;117(13):6976–84. doi: 10.1073/pnas.1921623117 (PMC7132279; doi:10.1073/pnas.1921623117)
Supplement: Supplementary File [file pnas.1921623117.sapp.pdf]

SUPPLEMENTAL INFORMATION

Box 1: Karl Lashley's Had a Broad Impact on the Study of Consciousness. Many researchers discussed in this paper are in Lashley's scientific family tree, including: Heinrich Kluver, Karl Pribram, Roger Sperry, Donald Hebb, Mortimer Mishkin, Brenda Milner, Michael Gazzaniga, Larry Weiskrantz, and Larry Squire).
Two of the authors of this paper are also scientific descendants of Lashley. J.E.L. is connected to him by way of Gazzaniga (who worked with Sperry), and also via Robert Thompson (who spent one year at Yerkes with Lashley (1)). H.L. is connected to Lashley by way of Richard Passingham, who worked with George Etlinger, who, in turn, worked with Lashley.

1. B. Michael Thorne (1995) Robert Thompson: Karl Lashley's heir? *Journal of the History of Behavioral Sciences.* 31: 129-136.

Box 2: Oher Patient Groups Also Contributed to Contemporary Understanding of Consciousness.

We focused on the contribution of amnesia, split-brain patients, and blindsight patients because of their broad impact on contemporary understanding of consciousness. But studies of other neurological patients contributed. For example, the discovery of the reticular activating system in animals in the 1940s began to provide an account of how transitions between wakefulness and sleep are regulated in the brain (1), and also provided insights into the loss of consciousness in coma patients (2, 3). Research on patients with so-called unilateral spatial neglect exhibited disturbances in the perception of visual space and alterations in conscious due to alterations in visual attention following damage to the parietal lobe (4, 5). Additionally, other patient groups (aphasia, dyslexia, and prosopagnosia) also exhibited dissociations between explicit knowledge and behavioral performance and have contributed to our understanding of consciousness (for review, see (6)).

1. Moruzzi G & Magoun HW (1949) Brain Stem Reticular Formation and Activation of the EEG. *Electroencephalography and Clinical Neurophysiology* 1:455-473.
2. Biemond A (1946) Diagnostiek van Hersenziekten. de Erven F. *Bohn, NV Haarlem*.
3. Plum F & Posner JB (1966) *Diagnosis of Stupor and Coma* (FA Davis, Philadelphia).
4. Paterson A & Zangwill OL (1944) Disorders of visual space perception associated with lesions of the right cerebral hemisphere. *Brain* 67(4):331-358.
5. Critchley M (1953) *The Parietal Lobes* (Edward Arnold, London).
6. Schacter D, McAndrews MP, & Moscovitch M (1988) Access to consciousness: dissociations between implicit and explicit knowledge in neuropsychological syndromes. *Thought without language*, ed Weiskrantz L (Oxford University Press, Oxford), pp 242-278.

www.pnas.org/cgi/doi/10.1073/pnas.1921623117

Box 3: Do Split-Brain Patients Have Two Conscious Minds?

Dual consciousness has been debated since the 1960s (1-4). Recently, the discussion was reinvigorated in the literature (5-8) and in the form of a debate at New York University.

A key issue not fully addressed by the critics of dual consciousness is that patients in the literature differ in a number of important ways: locus of their epileptic brain pathology; age of disease onset; age when the surgery was performed; how much of the corpus callosum was sectioned; whether other commissures were also sectioned; and how long after surgery they were tested. As a result, rather than asking the general question of whether dual consciousness exists in split-brain patients, it is more useful to address this question on a case-by-case basis. Cases described in the main text support the idea that two conscious minds can exist in the same head in some patients, especially when the right hemisphere has acquired some language processing skills. Whether right hemisphere language in necessary, or simply facilitates the assessment of consciousness, is not known.

1. Gazzaniga MS (1972) One brain - Two minds. *American Scientist* 60:311-317.
2. MacKay DM & MacKay V (1982) Explicit dialogue between left and right half-systems of split brains. *Nature* 295(5851):690-691.
3. Trevarthen C (1979) The tasks of consciousness: how could the brain do them? *Ciba Found Symp* (69):187-215.
4. Sperry R (1984) Consciousness, personal identity and the divided brain. *Neuropsychologia* 22(6):661-673.
5. Pinto Y*, et al.* (2017) Split brain: divided perception but undivided consciousness. *Brain* 140(5):1231-1237.
6. Volz LJ & Gazzaniga MS (2017) Interaction in isolation: 50 years of insights from split-brain research. *Brain* 140(7):2051-2060.
7. Rosen V (2018) One Brain. Two Minds? Many Questions. *J Undergrad Neurosci Educ* 16(2):R48-R50.
8. Schechter E (2018) *Self-Consciousness and 'Split' Brains: The Mind's I* (Oxford University Press, Oxford).
